# The Effect of Co-Culture with Different *Pichia kluyveri* and *Saccharomyces cerevisiae* on Volatile Compound and Characteristic Fingerprints of Mulberry Wine

**DOI:** 10.3390/foods13030422

**Published:** 2024-01-28

**Authors:** Bo Ding, Shutian Zhao, Wenxue Zhang, Ying Lin, Ling Xiong

**Affiliations:** 1 College of Biomass Science and Engineering, Sichuan University, Chengdu 610065, China; kelpbo@163.com (B.D.); zhaoshutian@stu.scu.edu.cn (S.Z.); 2022223080058@stu.scu.edu.cn (Y.L.); 2022223080061@stu.scu.edu.cn (L.X.); 2 School of Liquor-Brewing Engineering, Sichuan University of Jinjiang College, Meishan 620860, China

**Keywords:** mulberry wine, *S. cerevisiae*, *P. kluyveri*, co-fermentation, GC-IMS

## Abstract

In this study, changes in volatile compounds co-fermented by different *Pichia kluyveri* with *Saccharomyces cerevisiae* were analyzed using GC-IMS and compared with *S. cerevisiae* fermentation, to investigate the production of aroma in mulberry wine during the fermentation process. A total of 61 compounds were accurately identified, including 21 esters, 10 alcohols, 8 aldehydes, 6 ketones, and 19 other volatiles. Compared with the single strain fermentation (*S. cerevisiae*), the content of 2-methylpropyl acetate, allyl Isothiocyanate, ethyl crotonate, isobutyl propanoate, and butyl 2-methylbutanoate, co-fermentation groups (*S. cerevisiae* with different *P. kluyveri*) showed a significant decrease. Alcohols, aldehydes, ketones, and organic acid were lower in both the F(S-P1) and F(S-P2) groups than in the F(S) group throughout fermentation. The 2-methylpentanoic acid only was contained in the F(S) group. The co-fermentation with different *P. kluyveri* could also be well distinguished. The content of Benzaldehyde and 4-methylphenol in the F(S-P1) group was significantly lower than that in the F(S-P2) group. The PCA results revealed effective differentiation of mulberry wine fermented by different fermentation strains from GC-IMS. The result showed that *P. kluyveri* could establish a new flavor system for mulberry wine, which plays a crucial role in enhancing the flavor of fruit wine.

## 1. Introduction

Mulberry fruit (*Mores alba* L.) is one of the most popular fruits of the mulberry genus for their nutritional value, flavor, and ability to improve health [1,2]. Mulberry fruit is rich in nutrients including fatty acids, amino acids, vitamins, minerals, and bioactive compounds such as anthocyanins, rutin, quercetin, chlorogenic acid, and polysaccharides [3]. Due to the abundance of natural compounds it contains, previous studies have shown that mulberry fruit has anti-inflammatory, antioxidant, antibiotic, and hepatoprotective properties [4].

However, the storage and transport performance of fresh mulberries has not been satisfactory [5]. Plenty of mulberries are used for fresh consumption and primary processing, such as making juice, mulberry jam, and dried fruit [6]. To enrich mulberry products and adjust consumer needs, fruit wine, as a good value-added product, has gained increasing attention in recent years in China. Mulberry wine is one of the most popular fruit wines consumed worldwide [7,8]. As a traditional fermented alcoholic beverage with substantial territorial and socio-cultural significance, wine has a long history that is patterned by a tradition of innovation [9]. Presently, fruit wine, a different fruit-based fermented alcoholic beverage, has been accepted by people because of its low alcohol content and special flavor characteristics.

Generally, traditional fruit winemaking often involves fermenting a single yeast [10]. The *Saccharomyces cerevisiae* is a traditional ferment used to produce fruit wine because of its strong alcohol fermentation capacity [11]. However, single strain fermentation could make for a similar flavor and less characteristic profiles of the products. In recent years, non-*Saccharomyces* have received increasing attention. A cohort of scholars alternated to traditional fermentation performed by co-fermentation of *S. cerevisiae* and non-*Saccharomyces*, which lead to significant changes in the chemical composition and flavor characteristics of fruit wine [12]. Among the non-*Saccharomyces* yeasts, *Pichia kluyveri* has been considered a potential yeast in fruit wine processing because of its excellent aroma production ability. It has been reported that *P. kluyveri* increases the production of glycerol and esters [13]. And *P. kluyveri* could contribute to the production of aroma compounds, such as 2-phenylethyl acetate and 3-sulfanylhexan-1-ol acetate [14,15]. In addition, *Pichia fermentans*, either dead or alive, can make a positive contribution to promote the growth of *Oenococcus oeni*. And the co-fermentation with *Pichia fermentans*, improved the production of yeast-derived and grape-derived volatiles, which enhances the fresh fruity aroma of wines [16]. Therefore, it is believed that co-fermentation with *S. cerevisiae* and *P. kluyveri* could be a promising brewing method to introduce new flavors to mulberry wines. We intended to ferment mulberry juice by co-culturing of *S. cerevisiae* and *P. kluyveri*, thereby comparing the flavor differences of mulberry wine between single and co-culture fermentation.

For fruit wine, volatile compounds of flavor play a critical role in wine quality, which directly affects the quality and grade of wine. Typically, GC-MS (gas chromatography-mass spectrometry) can separate and identify volatile compounds from complex samples relatively accurately for the purpose of evaluating the flavor of products. However, the detection performance of GC-MS on low molecular weight (below 300 daltons) compounds is not ideal. Small molecules (C2–C10) with a low content of volatile components below 300 daltons, such as some ketones, acids, furans, and other compounds, can be key components in the modification of flavor. Previous studies have shown that these substances can be characteristic of different types of wine aroma. By monitoring these substances, the fermentation process can be precisely controlled. This will improve the quality of the product [17,18].

At present, GC-IMS (gas chromatography-ion mobility spectrometry) has a good detection effect on such small molecule volatile substances [19]. Ion mobility spectrometry (IMS) is an analytical technique to detect trace gases and to characterize chemical ionic substances based on the difference in the rate of migration of gas phase ions in an electric field [20]. The advantages of gas chromatography ion mobility spectrometry (GC-IMS) is that it requires no sample pretreatment, enables fast analysis timeframes, and has a low detection limit [21], which is a well-established technology that has been widely used in airport security, military security, and drug detection fields nowadays [22]. Especially, due to its lower testing and incubating temperature requirements, GC-IMS has promising prospects for food flavor analysis as it does not damage the original compounds of the food [23]. Previous research has confirmed that IMS is an effective method for the analysis and characterization of volatile compounds with different properties [24,25]. Many studies have also confirmed that GC-IMS technology minimizes loss of flavor substances, which is suitable for food volatile components analysis during storage [26] and processing [27].

Therefore, the aim of this study was to ferment mulberry juice by co-culturing *S. cerevisiae* and *P. kluyveri*, and monitor the release of VOCs in mulberry wine during fermentation; then, comparatively analyze the differences of the VOCs between single and co-culture fermentation; and finally, establish the characteristic volatile fingerprints of the VOCs in mulberry wine. On that basis, we can appraise the aroma influence of marker volatile composition in co-fermentation by difference strains so as to understand the immense potential of *P. kluyveri* in applications. At the same time, the creation of fingerprints of flavor compounds at different stages of fermentation will also contribute to the targeted regulation of flavor during the fermentation process.

## 2. Materials and Methods

### 2.1. Material

Healthy and ripe mulberries of “Yun No. 2” at commercial maturity were provided by Sixi Agricultural Development Co., Ltd. (Panzhihua, China). The strains *Saccharomyces cerevisia* (S), *Pichia kluyveri* (P1), and *Pichia kluyveri* (P2) were screened from the mulberry orchard (Panzhihua, China).

### 2.2. Mulberry Juice Fermentation

Squeezed and standardized mulberry juice (Brix, 25 °Bx). The strains *S*. *cerevisia* (S), *P. kluyveri* (P1), and *P. kluyveri* (P2) were separately pre-cultured in YPD medium at 28 °C with 170 rpm. After 40 h incubation, the yeast cells were separately collected, washed twice, and then resuspended in the 30 mL sterile saline solution. Finally, approximately 10^6^ CFU/mL of each yeast strain was transferred into the 500 mL of pasteurized mulberry juice. Fermentation tests were set up as follows: (1) F(S): single inoculation with *S. cerevisiae*, (2) F(S-P1): co-inoculation of *S. cerevisiae* (S) and *P. kluyveri* (P1), (3) F(S-P2): co-inoculation of *S. cerevisiae* (S) and *P. kluyveri* (P2), and the inoculum ratio was 1:1 [10]. We set the natural fermentation group as the control (CK). The control group and each fermentation broth were fermented at 16 °C for 18 days. Then, the samples were injected into the centrifuge tube and stored at −80 °C in frozen storage every 6 days (0, 6, 12, 18 days) to prepare for the subsequent analyses.

### 2.3. Methods

All samples were analyzed for the presence of volatile compounds using a gas chromatography-ion mobility spectrometry (GC-IMS) instrument (FlavourSpec^®^, Gesellschaft für Analytische Sensorsysteme mbH, Dortmund, Germany), which connected with a MXT-WAX capillary column (30 m × 0.53 mm × 1.00 um, RESTEK, Bellefonte, PA, USA). The n-ketones C4–C9 (Sinopharm Chemical Reagent Beijing Co., Ltd., Beijing, China) were used as external references to calculate the retention index (RI) of volatile compounds. The VOCs were identified by comparing the RI and the drift time (in milliseconds) of the standard in the GC-IMS library.

A total of 3 mL of mulberry wine was absorbed, then placed into a 20 mL headspace vial. Subsequently, samples were incubated at 45 °C with 500 rpm for 10 min. After incubation, 500 µL of volatile composition was automatically injected into the injector by means of a heated syringe at 50 °C with splitless mode. The oven temperature was set as 60 °C with isothermal conditions. The samples were driven into the column by nitrogen at a programmed flow as follows: 2 mL/min for 10 min, 15 mL/min for 5 min, 50 mL/min for 5 min, 100 mL/min for 5 min. Then, they were ionized in the IMS ionization chamber of 45 °C. The drift gas flow was set at a constant flow of 150 mL/min.

All analyses were performed in triplicate. Identification of volatiles were identified by comparing retention indices (RI) and the drift time (in milliseconds) of standard in the GC-IMS library [19].

### 2.4. Statistical Analysis

The instrumental analysis software LAV version 2.0.0, G.A.S. (Laboratory Analytical Viewer) and three attached plug-ins were used for sample analysis. Particularly, the program GC-IMS Library Search was applied to analyze the characteristic flavor substances. Then, the Reporter and Gallery plug-ins (G.A.S., Dortmund, Germany) were used to characteristic fingerprint the volatile flavor compounds, and the multivariate data analysis (PCA) was performed using the Dynamic PCA plug-in (G.A.S., Dortmund, Germany). Correlation analysis among volatile compounds was analyzed using Origin 2023 b software (Origin-Lab Corporation, Northampton, MA, USA).

## 3. Results and Discussion

### 3.1. GC–IMS Topographic Plots in Mulberry Wine during Fermentation

**Figure 1:**
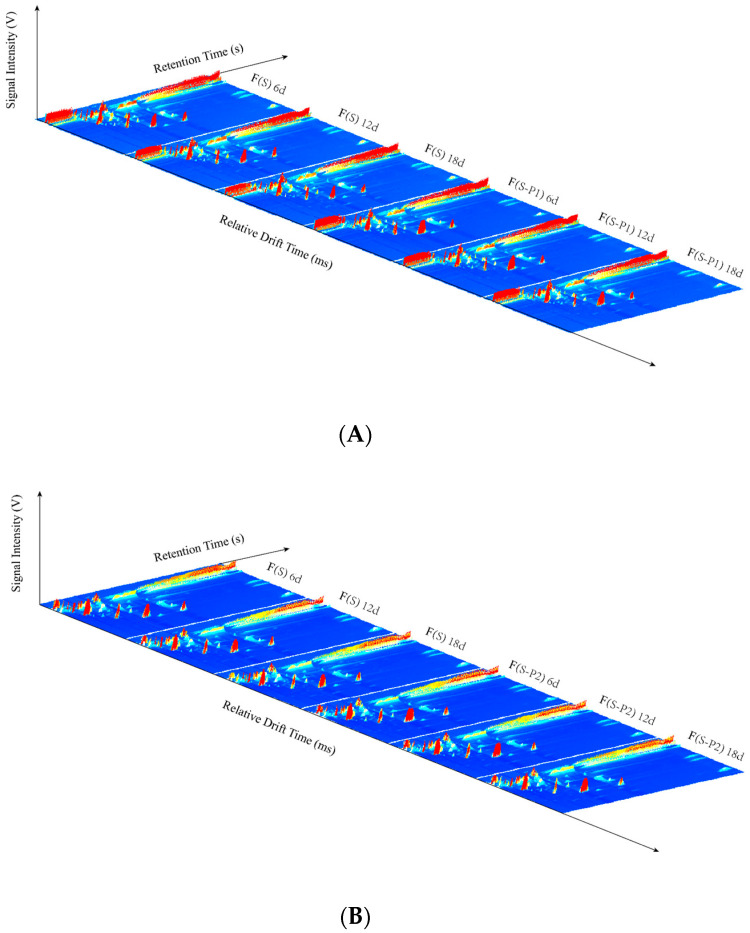

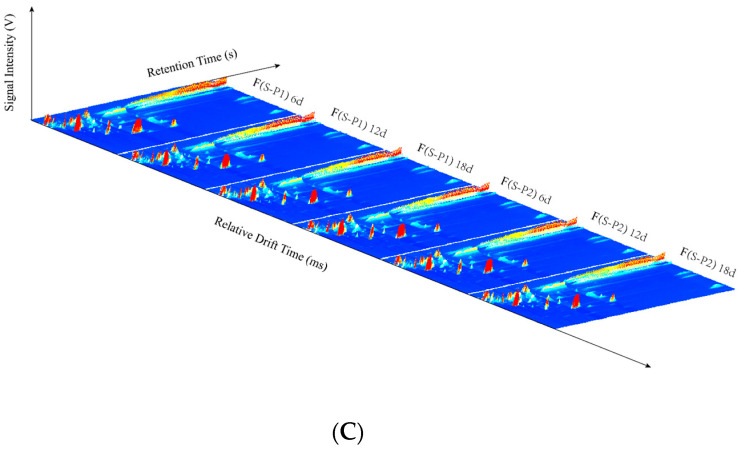
3D-topographic plots in different mulberry wine samples during fermentation. (**A**) group F(S); (**B**) group F(S–P1); (**C**) group F(S–P2).

**Figure 2:**
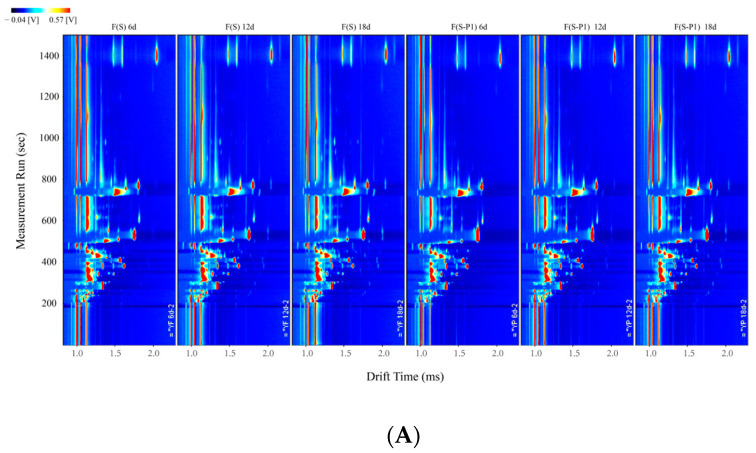

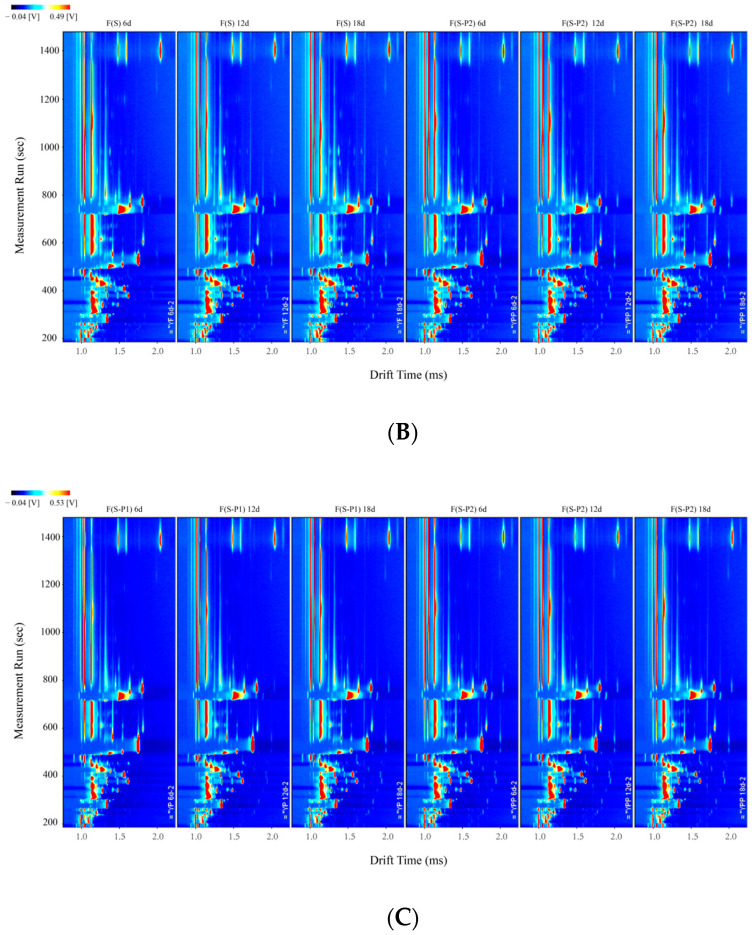
2D-topographic plots in different mulberry wine samples during fermentation. (**A**) group F(S) vs. group F(S-P1); (**B**) group F(S) vs. group F(S-P2); (**C**) group F(S-P1) vs. group F(S-P2).

The differences of volatile compounds in mulberry wine samples during the fermentation process were analyzed by GC-IMS. As shown in Figure 1, the GC-IMS analysis of sample volatiles resulted in a 3D-topographic plot, where *X*, *Y*, and *Z*-axes, respectively, represent the ion migration time (DT) for identification, the retention time (RT) of the gas chromatograph, and the peak intensity for quantification. The 2D-topographic spectra of volatile compounds during fermentation with treatment samples are shown in Figure 2.

In Figure 1 and Figure 2, the overall blue background was the GC-IMS spectra, and the remarkable red vertical line at abscissa 1.0 was the reactive ion peak (RIP) after normalization. Each heterochromatic or homochromatic point on both sides of the RIP line represented a volatile compound, which indicated the concentration of compound by different levels of color saturation. In general, light color represents lower concentration and redness reflects higher concentration, which means, the darker color indicates the higher concentration of the volatile compound [28]. The effect of fermentation with different strains on the volatile compounds is a significant difference. And there are also varying degrees of differences in peak intensity.

In Figure 2, all the mulberry wine samples are rich in volatile compounds. It can be seen that most of the peak signals appeared in the retention time of 100–900 s, and a few signals appeared in the retention time of 900–1400 s. The peak signals were observed in ranges of drift time of 1.0–2.0 ms.

**Figure 3:**
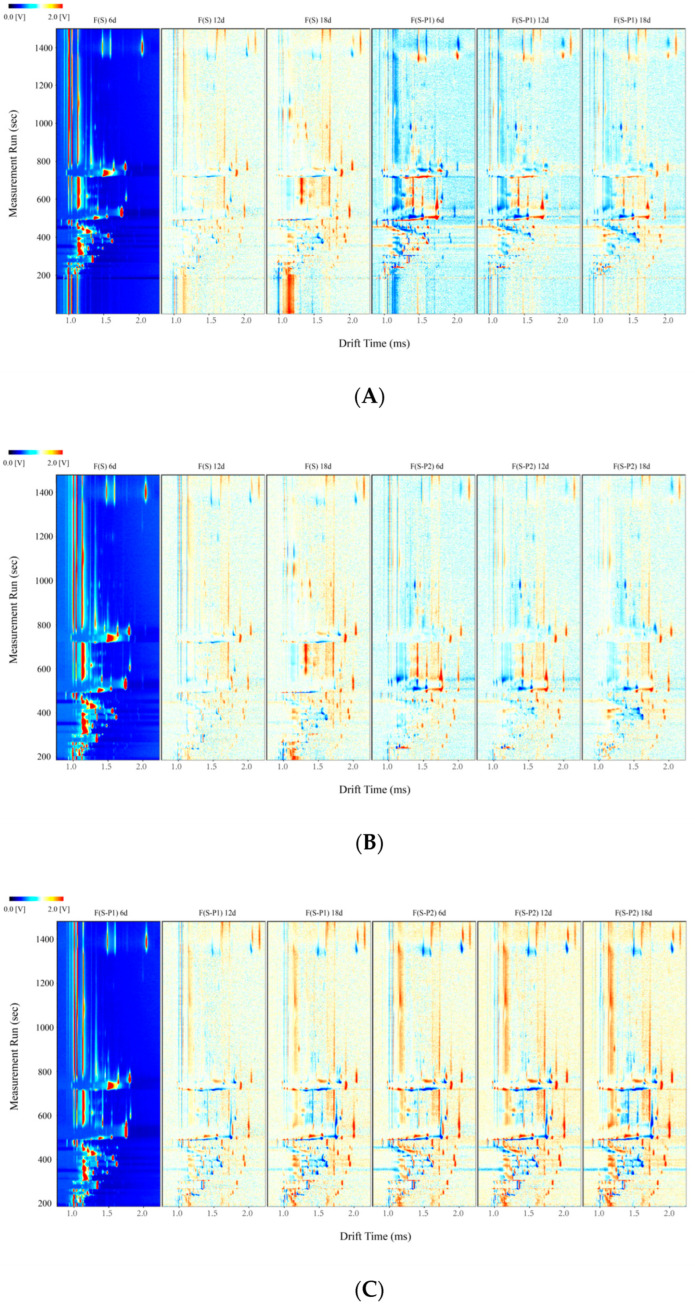
The comparison topographic plots in different mulberry wine samples during fermentation. (**A**) group F(S) vs. group F(S-P1); (**B**) group F(S) vs. group F(S-P2); (**C**) group F(S-P1) vs. group F(S-P2).

The difference comparison model was applied to compare the aroma differences among the samples, conveniently. The topographical plot of F(S) 6d was taken as the reference and the topographical plots of other samples (including F(S) 12d, F(S) 18d, F(S-P1) 6d, F(S-P1) 12d, and F(S-P1) 18d) were deducted from the reference (Figure 3A). For each dot after deduction appearing in the figure, the red color means a higher concentration of a volatile compound than the reference, blue color indicates a lower concentration of a volatile compound than the reference, and the white dot implies the volatile compounds are consistent with the reference. Similarly, Figure 3B also displayed on this principle, and F(S) 6d was taken as the reference as well. As shown in Figure 3, significant differences between F(S) and F(S-P1) groups were observed, which indicated the difference in molecule structure and concentration of volatile compounds [29]. This appearance should be caused by the participation of non-*Saccharomyces* (P1 and P2). Notably, there were also significant differences among the fermentation broth in different periods. As shown in Figure 3, some volatiles showed significant color differences in the spectra at different stages of fermentation. It is able to show the changes in the content of the substance as the fermentation process takes place.

### 3.2. Fingerprint Analysis of Volatile Compounds during the Fermentation Process of Mulberry Wine with Different Strains

**Figure 4:**
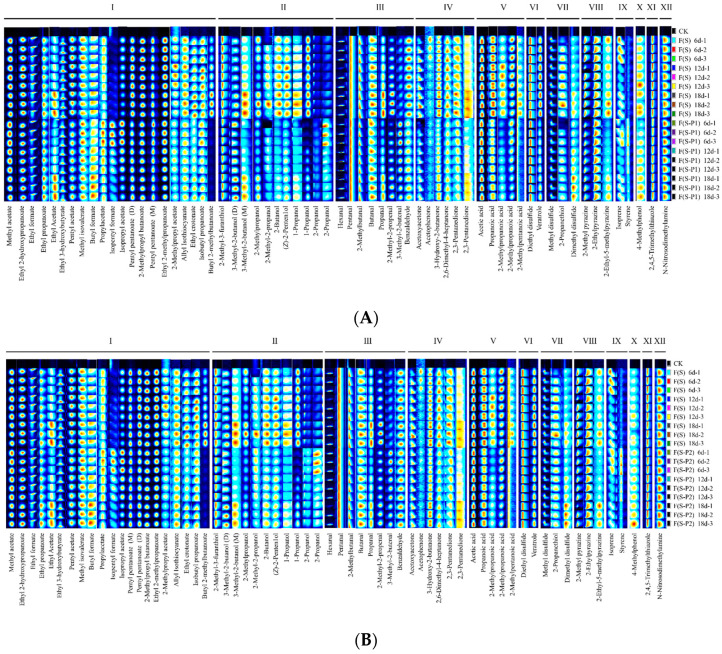

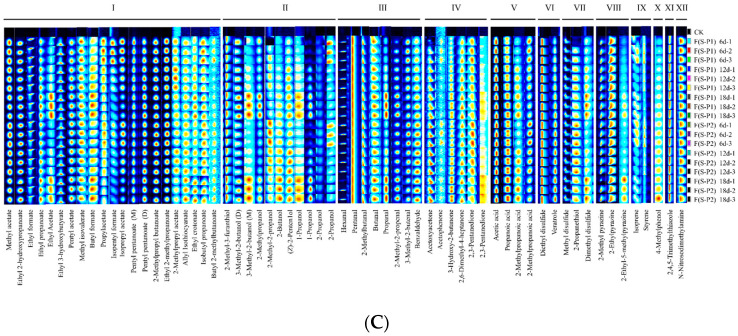
Fingerprints of volatile compounds in different mulberry wine samples during fermentation. (**A**) group F(S) vs. group F(S-P1); (**B**) group F(S) vs. group F(S-P2); (**C**) group F(S-P1) vs. group F(S-P2). I refers to esters, II refers to alcohols, III refers to aldehydes, IV refers to ketones, V refers to acids, VI refers to ethers, VII refers to sulpho compounds, VIII refers to pyrazine, IX refers to terpenes, X refers to phenol, XI refers to thiazole, and XII refers to nitrogen compound.

To further identify the differences of volatile compound profiles in different mulberry wines during fermentation, all information provided to qualitatively characterize by the fingerprint analysis technique. It means to give an overall profile of the aroma composition, not only based on the identification of one or two volatile compounds [30]. Therefore, the Gallery Plot plug-in could be used for previewing the volatile fingerprints by showing the color change trend of each point in the 2D plot, which included all volatile substances identified by the GC-IMS instrument (Figure 4). As shown in Figure 4, Gallery Plot listed volatile components together for intuitive comparison, all bright points of each row represented the entire signal peak of a sample, and each column showed the signal strength of the same substance detected in different periods of fermentation.

IMS drift times and retention indices were compared with authentic reference compounds to characterize these compounds. In Figure 4, generally, a total of 64 flavor substance signals were measured. Among these peaks, 61 compounds were accurately identified (based on the GC-IMS library, NIST database). Among these volatiles, as shown in Table 1, included 21 esters, 10 alcohols, 8 aldehydes, 6 ketones, and 19 other volatiles such as terpenes and acids (Table 1). It should be noted that there were 3 compounds, which had monomer (M) and dimer (D) form, produced two peak signals by one volatile (Figure 4 and Table 1).

The content of some ester substances in all experimental groups was relatively high in the early stage of fermentation, but showed an overall decreasing trend as fermentation progressed. That was probably because the rapid proliferation of yeast during fermentation contributes to the formation of esters [28,31]. Compared to the F(S) group, the F(S-P1) and F(S-P2) groups showed a significant decrease in this study. However, no significant difference was found between the F(S-P1) and F(S-P2) groups. As shown in Figure 4A, there were five identified ester substances, including 2-methylpropyl acetate, allyl Isothiocyanate, ethyl crotonate, isobutyl propanoate, and butyl 2-methylbutanoate. The F(S-P1) group substances were lower than those of the F(S) group during fermentation. It was also observed that the incorporation of *Pichia* (P1 and P2) had a greater effect on the formation of small molecule alcohols (Figure 4A,B). High levels of alcohol were observed in the F(S) group at different stages of fermentation. Alcohols were lower in both the F(S-P1) and F(S-P2) groups than in the F(S) group throughout fermentation. In this study, the levels of ester substances such as butyl 2-methylbutytanoate were negatively correlated with alcoholic substances. This phenomenon might be due to the fact that *Pichia* could significantly enhance the enzymatic reaction between higher alcohols and acetyl-CoA, thereby producing more esters [32]. Overall, the results of this study suggested that small molecule alcohols may also influence the formation of the corresponding esters. This was in line with the previous reports [28].

There were relatively few types of aldehydes and ketones in this study. Two types of carbonyl compounds gradually accumulated during the fermentation process. The F(S-P1) and F(S-P2) groups were significantly lower than the F(S) group. Benzaldehyde had a strong bitter almond and nutty aroma with a slight fruity sweetness [33]. There was no significant difference in the content of benzaldehyde between the F(S) group and the F(S-P2) group (Figure 4B), but the production of the F(S-P1) group was lower than that of the other groups throughout the fermentation process (Figure 4A,C). The results suggested that during the process of metabolism of *Pichia* (P1), the yield of benzaldehyde was consistently lower than that of the other two types of strain. In addition, a certain amount of benzaldehyde was produced during the metabolism of strain *S. cerevisiae* (S) itself (Figure 4A,B). The lower overall levels in the F(S-P1) group could also be due to the fact that the process of metabolism of *P. kluyveri* (P1) consumes a large amount of benzaldehyde, resulting in the absence of corresponding odor.

The organic acid substances F(S-P1) and F(S-P2) groups were significantly lower than the F(S) group, and there was no significant difference between the F(S-P1) and F(S-P2) groups. This could prevent the wine from developing an overly sharp acidic taste. But improper control could also make the wine taste monotonous. As shown in Figure 4C, most of the acids accumulated gradually with the fermentation process, while 2-methylpropanoic acid decreased gradually with the fermentation process. The content of 2-methylpropanoic acid in the F(S) group was consistently higher than that in the F(S-P1) and F(S-P2) groups. And the F(S-P1) group showed a significant decrease in content after 6 days, whereas the F(S-P2) group showed the same situation after 12 days. Notably, 2-methylpentanoic acid appeared only in the F(S) group and accumulated with fermentation. It did not appear in the F(S-P1) and F(S-P2) groups. It should be noted that the F(S) group has a distinct grassy taste compared to the other two groups in this study. The 2-methylpentanoic acid was probably partly responsible for this significant difference. This result is also consistent with previous reports [34].

The content of other substances, including ethers, pyrazines and others, was not significantly different among the experimental groups, and there was little change during the fermentation process. These substances were mostly formed in the early stages of fermentation and their levels did not change significantly as the fermentation process progresses (Figure 4A–C). It is noteworthy that both the F(S-P1) and the F(S-P2) groups showed a certain amount of dimethyl disulfide at the late stage of the fermentation process. Sulfur-containing substances, such as dimethyl disulfide, have an odor of their own. It would bring the special bouquet that the sulfur-containing substances had the right amount. However, its high levels could be very detrimental to fruit wines [35].

In addition, the only phenolic substance 4-Methylphenol is described as caramel flavor (threshold, 20 μg/L), which may become a potential hazardous substance [36]. In this study, 4-methylphenol levels remained consistently low in the F(S-P1) group (Figure 4A,C). It was higher in the F(S-P2) group than in the F(S) and F(S-P1) groups (Figure 4B,C). The results indicated that *Pichia* (P1) had a better inhibitory effect on the production of 4-methylphenol.

### 3.3. PCA Analysis of Different Mulberry Wine during Fermentation

**Figure 5:**
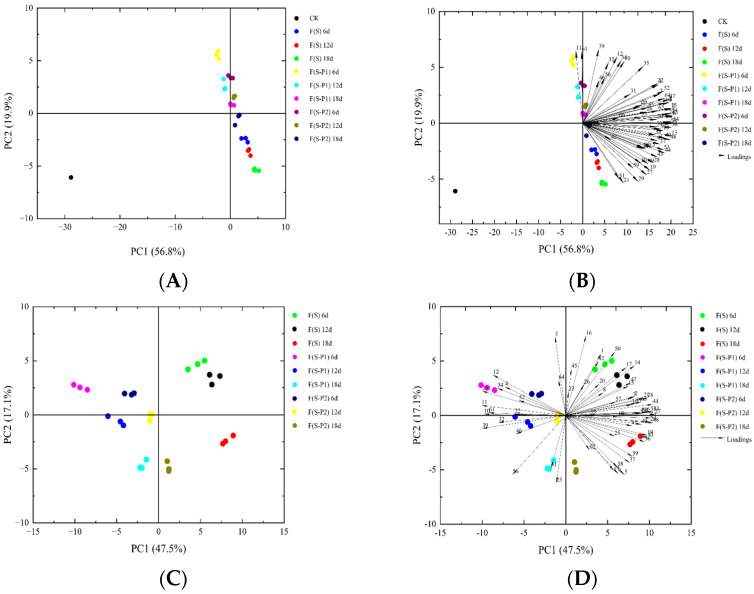
PCA analysis of flavor compounds in mulberry wine with different strains during the fermentation process. (**A**,**B**) The score plot and biplot of PCA analysis (with CK). (**C**,**D**) The score plot and biplot of PCA analysis (without CK).

In order to distinguish and present the differences among the different mulberry wine samples during fermentation, the principal component analysis (PCA) technique was used to evaluate the differences. In this study, the signal intensity of different volatile compounds identified by GC-IMS was used to structure the PCA model. The PCA of volatile compounds in different mulberry wine samples during fermentation is presented in Figure 5. The distribution map indicated that the total contribution ratio of the first two principal components was 64.6%, which collected 47.5% (PC 1) and 17.1% (PC 2) of the cumulative variance contribution rate. The PCA results exactly indicated that the fermentation with single and co-culture of yeast would be distinguished in the distribution map. The same sample also showed significant stage differences at different stages of fermentation. As show in Figure 5, all samples of F(S) could be separated by the positive score values of PC1, and the F(S-P1) group could be distinguished according to the negative scores of PC1. On the other hand, the score values of PC2 could be used to separate the different fermentation stages of fruit wine. On this foundation, together with biplot of PCA analysis, the result showed that the non-*Saccharomyces Pichia kluyveri* P1 and P2 could enhance the characteristic flavor of mulberry wine by improving the composition and content of characteristic aroma compounds, leading to the creation of new aromatic mulberry wines. As a result of differential metabolism of non-Saccharomyces, it would provide flavors with different characteristics. This also results in significant differences in wine flavor type.

## 4. Conclusions

Aroma is one of the most important means of evaluating and identifying a fruit wine. Fruit wines are frequently made with a particular type of *Saccharomyces cerevisiae*, which means that the raw materials significantly impact the flavor of the product. It should be noted that traditional methods are advantageous in molding product flavour, but the extension of fruit wine varieties is significantly restricted. Appropriate application of non-*Saccharomyces* could help fruit wines develop new and more attractive flavors. In this study, a total of 64 signal peaks from topographic plots were identified in different mulberry wine samples. The specificity of *P. kluyveri* (P1, P2) in regulating the production of 2-methylpentanoic acid, benzaldehyde and, conversely, 4-methylphenol is reported for the first time in this article, and deserves further investigation in future research. Additionally, the VOCs fingerprint also demonstrates the process of change in the content of different volatile substances at different stages of fermentation. In addition, the results of PCA showed that the different mulberry wines were well distinguished. Therefore, non-*Saccharomyces* will provide more options for creating fruit wine flavors and has great research potential for producing new types of fruit wine.

By constructing effective fingerprints, key flavor compounds can be identified. This allows rapid differentiation of the flavor characteristics of wines fermented by different non-*Saccharomyces*. This feature can be used to create fingerprint maps during fermentation to monitor changes in the levels of the key flavor compounds. It also means that by regulating the fermentation process based on changes in key flavor compounds, the quality of wine can be improved.

## Figures and Tables

**Table 1:** GC–IMS integration parameters of volatile compounds obtained from three different mulberry wine samples.

No.	Compound	CAS	Molecule Formula	MW ^a^	RI ^b^	RT ^c^ [s]	DT ^d^ [ms]	Comment
Ⅰ Esters								
1	Methyl acetate	79-20-9	C_3_H_6_O_2_	74.1	545	250.986	1.2007	
2	Ethyl 2-hydroxYPPropanoate	97-64-3	C_5_H_10_O_3_	118.1	811.2	505.891	1.552	
3	Ethyl formate	109-94-4	C_3_H_6_O_2_	74.1	622.9	303.678	1.2087	
4	Ethyl propanoate	105-37-3	C_5_H_10_O_2_	102.1	715.7	387.553	1.1441	
5	Ethyl Acetate	141-78-6	C_4_H_8_O_2_	88.1	562	261.299	1.0895	
6	Ethyl 3-hydroxybutanoate	5405-41-4	C_6_H_12_O_3_	132.2	950.1	762.614	1.6458	
7	Pentyl acetate	628-63-7	C_7_H_14_O_2_	130.2	932.1	722.363	1.3195	
8	Methyl isovalerate	556-24-1	C_6_H_12_O_2_	116.2	767	446.34	1.2081	
9	Butyl formate	592-84-7	C_5_H_10_O_2_	102.1	746.3	421.426	1.2121	
10	Propylacetate	109-60-4	C_5_H_10_O_2_	102.1	717.3	389.246	1.4832	
11	Isopentyl formate	110-45-2	C_6_H_12_O_2_	116.2	775.9	457.729	1.6333	
12	Isopropyl acetate	108-21-4	C_5_H_10_O_2_	102.1	670.6	343.527	1.489	
13	Pentyl pentanoate	2173-56-0	C_10_H_20_O_2_	172.3	1146.9	1407.006	1.4827	Monomer
14	Pentyl pentanoate	2173-56-0	C_10_H_20_O_2_	172.3	1145.7	1401.398	2.0471	Dimer
15	2-methylpropyl butanoate	539-90-2	C_8_H_16_O_2_	144.2	954.5	773.001	1.8113	
16	Ethyl 2-methylpropanoate	97-62-1	C_6_H_12_O_2_	116.2	736.4	410.037	1.5711	
17	2-Methylpropyl acetate	110-19-0	C_6_H_12_O_2_	116.2	745.4	420.316	1.6156	
18	Allyl Isothiocyanate	1957/6/7	C_4_H_5_NS	99.2	878.7	615.893	1.3861	
19	Ethyl crotonate	623-70-1	C_6_H_10_O_2_	114.1	846.4	560.062	1.5493	
20	Isobutyl propanoate	540-42-1	C_7_H_14_O_2_	130.2	842.2	553.34	1.7146	
21	butyl 2-methylbutanoate	15706-73-7	C_9_H_18_O_2_	158.2	1032	980.844	1.385	
Ⅱ Alcohols								
22	2-Methyl-3-furanthiol	28588-74-1	C_5_H_6_OS	114.2	861.1	584.731	1.1402	
23	3-Methyl-2-butanol	598-75-4	C_5_H_12_O	88.1	677.8	350.056	1.238	Monomer
24	3-Methyl-2-butanol	598-75-4	C_5_H_12_O	88.1	673.3	345.996	1.4334	Dimer
25	2-methylpropanol	78-83-1	C_4_H_10_O	74.1	616	298.43	1.3799	
26	2-Methyl-2-propanol	75-65-0	C_4_H_10_O	74.1	509.6	231.177	1.1317	
27	2-Butanol	78-92-2	C_4_H_10_O	74.1	610.1	294.048	1.1509	
28	(Z)-2-Penten1ol	1576-95-0	C_5_H_10_O	86.1	751.2	427.121	1.4368	
29	1-Propanol	71-23-8	C_3_H_8_O	60.1	531.3	243.032	1.1178	
30	1-Propanol	71-23-8	C_3_H_8_O	60.1	558.9	259.421	1.1108	
31	2-Propanol	67-63-0	C_3_H_8_O	60.1	531.6	243.228	1.1779	Monomer
32	2-Propanol	67-63-0	C_3_H_8_O	60.1	536.4	245.931	1.2186	Dimer
Ⅲ Aldehydes								
33	Hexanal	66-25-1	C_6_H_12_O	100.2	805.1	497.264	1.2544	
34	Pentanal	110-62-3	C_5_H_10_O	86.1	689.2	360.844	1.1745	
35	2-Methylbutanal	96-17-3	C_5_H_10_O	86.1	678.4	350.696	1.4006	
36	Butanal	123-72-8	C_4_H_8_O	72.1	610.1	294.048	1.1133	
37	Propanal	123-38-6	C_3_H_6_O	58.1	468.6	211.079	1.1393	
38	2-Methyl-2-propenal	78-85-3	C_4_H_6_O	70.1	572.1	267.793	1.2092	
39	3-Methyl-2-butenal	107-86-8	C_5_H_8_O	84.1	775.9	457.729	1.3505	
40	Benzaldehyde	100-52-7	C_7_H_6_O	106.1	960.5	787.283	1.4573	
Ⅳ Ketones								
41	Acetoxyacetone	592-20-1	C_5_H_8_O_3_	116.1	488.5	220.495	1.0945	
42	Acetophenone	98-86-2	C_8_H_8_O	120.2	1095.1	1195.186	1.5748	
43	3-Hydroxy-2-butanone	513-86-0	C_4_H_8_O_2_	88.1	737	410.749	1.3244	
44	2,6-Dimethyl-4-heptanone	108-83-8	C_9_H_18_O	142.2	972.2	815.848	1.3218	
45	2,3-Pentanedione	600-14-6	C_5_H_8_O_2_	100.1	689.8	361.451	1.2115	
46	2,3-Pentanedione	600-14-6	C_5_H_8_O_2_	100.1	710.9	382.528	1.217	
Ⅴ Acids								
47	Acetic acid	64-19-7	C_2_H_4_O_2_	60.1	600.8	287.345	1.341	
48	Propanoic acid	79-09-4	C_3_H_6_O_2_	74.1	711.4	382.987	1.3465	
49	2-Methylpropanoic acid	79-31-2	C_4_H_8_O_2_	88.1	769.8	449.899	1.3746	
50	2-Methylpropanoic acid	79-31-2	C_4_H_8_O_2_	88.1	746.9	422.138	1.3906	
51	2-Methylpentanoic acid	97-61-0	C_6_H_12_O_2_	116.2	1031.6	979.77	1.2668	
Ⅵ Ethers								
52	Diethyl disulfide	110-81-6	C_4_H_10_S_2_	122.2	916.8	689.903	1.1448	
53	Veratrole	91-16-7	C_8_H_10_O_2_	138.2	1146.3	1404.202	1.5962	
Ⅶ Sulpho compound								
54	Methyl disulfide	624-92-0	C_2_H_6_S_2_	94.2	771.5	452.035	1.1459	
55	2-Propanethiol	75-33-2	C_3_H_8_S	76.2	562.5	261.649	1.1434	
56	Dimethyl disulfide	624-92-0	C_2_H_6_S_2_	94.2	741.7	416.084	1.1397	
Ⅷ Pyrazine								
57	2-Methyl pyrazine	109-08-0	C_5_H_6_N_2_	94.1	808.2	501.576	1.3943	
58	2-Ethylpyrazine	13925-00-3	C_6_H_8_N_2_	108.1	938.5	736.295	1.5252	
59	2-Ethyl-5-methylpyrazine	13360-64-0	C_7_H_10_N_2_	122.2	1004.9	901.848	1.2119	
Ⅸ Terpenes								
60	Isoprene	78-79-5	C_5_H_8_	68.1	524.7	239.328	1.2107	
61	Styrene	100-42-5	C_8_H_8_	104.2	905.9	667.742	1.431	
Ⅹ Phenol								
62	4-Methylphenol	106-44-5	C_7_H_8_O	108.1	1069.9	1104.14	1.142	
Ⅺ Thiazole								
63	2,4,5-Trimethylthiazole	13623-11-5	C_6_H_9_NS	127.2	1002.6	895.425	1.1343	
Ⅻ Nitrogen compound								
64	N-Nitrosodimethylamine	62-75-9	C_2_H_6_N_2_O	74.1	755.5	432.25	1.2705	

^a^ MW: molecular mass; ^b^ RI: retention index; ^c^ RT: retention time; ^d^ Dt: drift time.

## Data Availability

Data is contained within the article.
